# Challenges faced by parents of children with learning disabilities in Opuwo, Namibia

**DOI:** 10.4102/ajod.v6i0.283

**Published:** 2017-07-26

**Authors:** Clever Taderera, Herna Hall

**Affiliations:** 1 Department of Social Work and Criminology, University of Pretoria, South Africa

## Abstract

**Background:**

Parenting children with learning disabilities requires a high level of knowledge and access to resources, information and services. In developing countries, however, these resources and services are not always available. Parents in Namibia, a developing country, therefore face challenges addressing children’s learning and other developmental disabilities, including challenges related to preventative and supportive interventions.

**Objective:**

This research focuses on challenges faced by parents as they parent children with learning disabilities in Opuwo, Namibia.

**Method:**

In-depth interviews were conducted with eight parents regarding the challenges they face in parenting their children with learning disabilities. Thematic analysis enabled the researchers to identify, analyse and report on themes that emerged from the qualitative interview data.

**Results:**

Analysis of the interviews indicated that some participants had only a vague understanding of learning disabilities, as they did not have access to essential knowledge about this phenomenon. They also lacked an awareness of the availability of programmes, services and policies meant to benefit their children with learning disabilities. Participants voiced that they, their children with learning disabilities and community members have stereotypes and prejudices regarding learning disabilities. In this study, most of the children with learning disabilities were raised by single, unemployed parents who seemed to have access to less support from external sources than married couples parenting children with learning disabilities. These single parents are usually not married and because of lack of financial support from the other parent, the majority of them indicated that they struggle to meet the financial and material needs of their children.

**Conclusion:**

The researchers concluded that the participants in this study experience a range of challenges in parenting their children with learning disabilities. The main challenges emanate from financial instability, as well as lack of knowledge regarding services and programmes for children with learning disabilities. This lack of knowledge on the part of participants could indicate poor policy education by policy implementers at grass-roots level.

## Introduction

When parents learn that their child has a learning disability (LD), ‘they begin a journey that takes them into a life that is often filled with strong emotions and difficult choices’ (Kalek 2008:20). Flack (2005:318) mentions inherent difficulties in defining learning disabilities, but presents ‘learners with special educational needs’ and what she describes as ‘the more in vogue, learners with barriers to learning’ as possible definitions. In the African context, the term learning ‘disabilities’ refers to children who experience learning challenges without presenting with obvious physical disabilities and who struggle with comprehension to a greater extent than the average child (Abosi 2007).

Namibia as a developing, low-income country faces challenges in the areas of preventing LDs as well as in terms of the inclusion of children with LDs in mainstream society. The National Policy for Mental Health (Republic of Namibia 2005) indicates that challenges faced by parents of children with LDs are exacerbated by factors such as community attitudes, cultural beliefs as well as institutional challenges. Because of the superstition that evil spirits, witchcraft or a mother’s improper relationships cause learning disabilities, women are often abandoned by their husbands and have to bear the brunt of caring for the child with LDs alone (Burke 2008; Haihambo & Lightfoot 2010; Harper et al. 2013). Haihambo and Lightfoot (2010) also report that among some African ethnic groups, fathers often desert their families when a child with a disability is born.

Opuwo, the capital of the Kunene region, is the geographical location of this study. The indigenous inhabitants, the OvaHimba people, practise a semi-nomadic lifestyle. Opuwo is considered one of the remotest areas in the country, with the lowest literacy rate of 58% among the adult population (15 years and above) (Republic of Namibia 2012b). School attendance is interrupted by the nomadic lifestyle. However, the government has introduced mobile schools that are established according to the movement patterns of the rural inhabitants to address high levels of illiteracy (Hailombe 2011). Nationally, Namibia is one of the countries with high levels of inequality with a Gini coefficient of 0.58 (Republic of Namibia 2012a). The child poverty rate in Namibia is 34.4%, while extreme household poverty is 10.3% (Republic of Namibia 2016). The government through its Integrated Early Childhood Development Policy (IECD) has programmes such as the family visitors’ programme which is a child-focused education programme offered by educarers, with a view to provide comprehensive support to children. This programme specifically promotes positive early childcare practices among previously socially disadvantaged groups in rural areas such as the OvaHimba, by educating them on antenatal care, nutrition and sanitation (Republic of Namibia 2007:4).

## Literature review

In this study, *parents* are defined as persons who are in custody of and take a series of actions to promote the development of a child (Musweu 2009 in Grobler 2012). *Parenting* is therefore the process of promoting and supporting the physical, emotional, social, spiritual and intellectual development of a child from infancy to adulthood (Mumbuna 2010). Burke (2008) postulates that parents do not necessarily have to be a couple caring for the child together, but could be a single parent.

Abosi (2007) argues that *learning disability* is a Western-based concept and that there are no indigenous conceptualisations to determine or diagnose LDs in Africa. Cortiella (2011) and Atkinson (2010) state that the major causes of neurological disorders are unclear, although LDs can be triggered by various factors such as prenatal and birth problems. However, what do seem to be clear are the cross-cutting challenges that are inherent in parenting a child with learning disabilities (Harper et al. 2013). The researchers will subsequently discuss the emotional challenges experienced by caregivers, attitudinal and cultural challenges and challenges with resources and services. This discussion will commence with the social model of disability as the theoretical framework for the research. Oliver (2004), one of the early exponents of the social model of disability, describes it as an instrument to gain insight into the manner in which society disables people with impairments. The social model of disability arose as a response to the critique of the medical model of disability. The medical model views individuals with disabilities as ‘the problem’, which indirectly denies opportunities to people with disabilities, restricts choice, self-determination and control over support systems in the lives of persons with disabilities. Oliver (2004) argues that, according to the medical model, individuals need to be ‘fixed’, which promotes dependency, charity and sympathy. In contrast, the social model of disability forms the basis of a rights-based approach to disability as it promotes independence and stimulates potential in persons with disabilities. Booyens, Van Pletzen and Lorenzo (2015) argue that family attitudes and behaviour prevent persons with disabilities from developing to their full potential. Promoting independence and stimulating potential in persons with disabilities proposes the opposite, thereby endorsing the social model of disability.

The social model of disability posits that at the root of disability and disablement are sociopolitical constructs. Hence, disability movements utilise the social model of disability as a political platform and tool to advocate for the rights of disabled people, with the objective of ensuring that they enjoy the status of full citizenship within contemporary society (Swain et al. 2004). The social model of disability is also relevant and an integral part in achieving social development because it enables the building of social capital by emphasising the removal of attitudinal and cultural barriers to persons with disabilities (Oliver 1990).

Harper et al. (2013) point out that there are wide-ranging challenges that are integral to parenting a child with LDs. Children with learning disabilities generally require more attention because they may have additional needs when compared to nondisabled children (VanPelt 2007). Grobler (2012) mentions that parents of children with LDs may experience constant subjection to a guilty feeling that they may be directly responsible for the disability through genetics, stressful moments while pregnant or abuse of alcohol. A study conducted in the USA by Resch et al. (2010) revealed that because of increased parental responsibilities, parents of children with LDs can be at a high risk of experiencing depression, physical health problems and decreased quality of life. In Namibia, these challenges could be exacerbated by extreme household poverty (Republic of Namibia 2016). Understanding the beliefs and myths about parenting further helps to explain why parents of children with disabilities are engulfed with emotional stress. In Namibia, there is a general expectation that children ‘bring happiness to a marriage’ and that womanhood is proven by giving birth to a ‘normal’ child (Chilwalo 2010:19). Grobler (2012) lists some of the common myths about parenting in Namibia: having a child will save an unhappy marriage, to have a baby will solve emotional problems and to have a baby is a romantic experience. These high expectations often turn into some level of disappointment if the child born has impairments, as some communities in Namibia perceive a child with impairments as a curse from God and an unfaithful wife is regarded as the cause of the disability (Chilwalo 2010). These examples illustrate how attitudes towards disability may impact parents’ experiences and challenges of caring for children with disabilities. Proponents of the social model of disability put forward that even though an impairment exists, its significance should be neutral, neither negative nor positive, as disability is the outcome of a complex relationship between a health condition and personal and external factors, such as the circumstances in which the individual lives. The social model of disability focuses on eliminating societal barriers by promoting the rights of persons with disabilities (Harries & Enfield 2003).

Meekosha (2011:667) postulates that the majority of research conducted in this field has been based on Western experiences, while very few have focused on third-world experiences, particularly within the African context. Research has however been conducted on LD and disability in general in various African countries such as South Africa (Mohamed & Laher 2012; Mudhovozi, Maphula & Mashamba 2012), Tanzania (McNally & Mannen 2013), Swaziland (Thwala, Ntinda & Hlanze 2015), Zimbabwe (Van der Mark & Verrest 2014) and Kenya (Gona et al. 2011). It is indicated in all these studies that children with disabilities in Africa are subject to belittling and offensive name calling. Abosi (2007) points out that children with LDs specifically are subject to derogatory and demeaning names such as ‘slow learners’ or ‘underachievers’. The authors have noted that in Opuwo children with LDs also have demeaning names such as ‘ejova’, translated into English as ‘stupid’, or ‘otjirengeona’ meaning not a perfect or complete human being. These beliefs culminate in stigmatisation and discrimination against the child or families of a child with LDs. Abosi (2007) adds that generally because of cultural beliefs about disabilities, some communities perceive disabilities as a punishment for what one has done wrong. This view is supported by various researchers (Masasa, Irwin-Carruthers & Faure 2005; Mudhovozi et al. 2012; Thwala et al. 2015) who refer to witchcraft, punishment by God, curse from the gods and a sign of bad omen. However, Mathye and Eksteen (2016:591) do not emphasise punishment, but rather the ‘Will of God’ as a reason for disability.

Because of lack of accurate information regarding LDs, extended family members may be unwilling to contribute to and support the raising of a child with LDs for fear of associated discrimination and stigmatisation. Bayat (2014:3) describes two notions that cause resentment towards children with disabilities. Firstly, the Western Judeo-Christians perceive creation as being ‘perfect’ or ‘normal’ and thus any deviation from normalcy is seen as evil or the result of sin. Secondly, African indigenous religions explain the world in a context of interaction between various natural spirits: sky, water, forest, earth and ancestors. Problems and disharmony in life often means two or more spirits are in conflict. Illness or affliction is considered to be the result of displeasure of one of the natural or ancestral spirits or a result of possession of an evil spirit (Bayat 2014).

Because of the existence of both these religious and cultural beliefs in Africa, a family that gives birth to a child with a disability is subject to social stigma and discrimination. In some instances, as noted by Harper et al. (2013), marriages may fail because of accusations of evil spirits when a child with any form of disability is born. Traditionally, communities in Namibia are organised on patriarchal lines (LaFont & Hubbard 2007). Hence, women are usually the accused in the event of an unsuccessful marriage and as such they bear the responsibility of parenting children with disabilities as lone parents (Chilwalo 2010). Challenges in parenting children with LDs are also encountered because of lack of resources, especially resources related to daily living support. Resch et al. (2010) argue that when parents have the perception that their situation (caring for the child with LDs) exceeds support at their disposal in terms of resources, it becomes a burden. This phenomenon is not unusual in Namibia seeing that extreme household poverty is 10.3% (Republic of Namibia 2016).

In Namibia, the lower levels of education, particularly in the Kunene region where the study was conducted, may make it more difficult for parents of children with LDs to access information on the needs of their children as some information can be accessed through technology such as the Internet and social media platforms. The position paper on Youth Education and Skills Development (Republic of Namibia 2012b) indicates that of the 14 regions in Namibia, the Kunene region has the lowest literacy rate of 58% among the adult population. Therefore, although information and resources might be available through private entities, non-governmental organisations as well as government ministries, the low level of literacy may be a barrier for parents to effectively engage with service providers.

Financial hardship is another challenge that parents of children with LDs face. Resch et al. (2010) argue that in general, families of children with disabilities, regardless of the type of disability, experience higher expenditure than other families. Freedman and Boyer (2000) also note that exacerbating these financial challenges is the finding that children with disabilities are significantly more likely to live in families considered to be poor. A possible explanation could be that disadvantaged communities may lack adequate resources for healthcare, experience poor access to vital information on the well-being of families, as well as low educational levels. Lukemeyer, Meyers and Smeeding (2000) support this view by pointing out that children in low-income families are more likely to suffer disabilities than more affluent families, while a child’s disability might also increase the risk of the family being poor. Park, Turnbull and Rutherford Turnbull (2002:160) make the statement that ‘poor families of children with a disability will be affected by poverty more severely than either poor families of nondisabled children or affluent families of children with a disability’. In Namibia, adverse conditions such as poor antenatal care, nutrition and sanitation hinder the early detection and prevention of impairments in children (Republic of Namibia 2007). Landman and Lombard (2006) argue that the focus on individuals and the family within a developmental approach include employing a capacity-building, strengths perspective and effecting structural changes within the community. The Government of the Republic of Namibia is championing the development of an integrated social development policy that will set the foundation for an efficient social development welfare system in Namibia (Republic of Namibia 2014, in Chiwara 2015). Chiwara (2015) concludes that through a combination of social and economic goals, the well-being of society as a whole lies at the heart of social development. In the Namibian context, social workers can engage families of children with disabilities in a number of ways that can enhance their resource base. One such way is empowering parents to establish small enterprises that can generate income that will help them meet their and their children’s needs. The researchers are of the view that social workers could also engage these parents in community-based rehabilitation committees (CBRCs) where they can be empowered with information regarding disabilities, as well as in networking with organisations for people with disabilities to enhance self-help, peer support and community-based projects. Parents of children with disabilities face the challenge of fragmented services as children’s services in Namibia are provided by different ministries (Republic of Namibia 2011). The Ministry of Gender Equality and Child Welfare deals with promotion of the rights of orphans and vulnerable children; the Women and Child Protection Unit under the Ministry of Safety deals with investigations of abused children; the Ministry of Labour and Social Welfare administers social grants that children with disabilities and orphans are entitled to; the Ministry of Health and Social Services, through the medical officers, approves or disapproves the applications for social grants, both for adults and children (Republic of Namibia 2011). The fragmentation of these services may pose challenges to those who want to access them. Parents, through the Namibia Planned Parenthood Association, voiced the concern that the official procedures in accessing social grants make it difficult for them to apply for grants for their children (Namibia Planned Parenthood Association 2013). In summary, apart from frustrations in accessing services, the parents of children with disabilities also face challenges in terms of beliefs and myths about parenting in general and about parenting children with impairments. This leads to the risk of experiencing emotional and physical health problems. Emotional stress is further exacerbated by poverty, lack of resources or a lack of knowledge regarding available resources. Advocating for the removal of attitudinal barriers, promoting independence and stimulating potential in persons with disabilities, as proposed by the social model of disability, will promote the right of persons with disabilities.

## Objectives

The goal of this study was to explore and describe the challenges faced by parents of children with learning disabilities in Opuwo, Namibia. As social workers, the researchers have an interest in the social functioning of such parents. Challenges faced have a direct influence on social functioning. Specific objectives were to (1) contextualise learning disabilities as well as parenting as phenomena by reviewing the literature, (2) to explore and describe the challenges of parenting children with learning disabilities from the perspectives of the parents in this study and (3) to make recommendations for practitioners regarding the experiences of parents who parent children with learning disabilities in similar contexts.

## Research methodology

In this study the researchers aimed to obtain an in-depth understanding of the experiences of parents in parenting children with LDs. Based on Kumar’s (2011) characteristics of qualitative research, namely an unstructured, flexible and open approach to enquiry, this study was qualitatively rooted in order to obtain an in-depth understanding of these experiences.

Information regarding personal challenges in raising a child with LDs may be sensitive in nature and affect individuals on a personal level. Therefore, the researchers utilised a phenomenological research design in order to obtain the meaning of these personal challenges as experienced by parents. This design allowed parents to reflect on and share their life world as it relates to the learning disabilities of their children (Fouché & Schurink 2011). Creswell (2007) as cited in Fouché and Schurink (2011) states that at the root of phenomenology is the intent to understand the phenomenon under study and therefore to provide a description of lived experiences by multiple subjects. The study population was all the parents of children with LDs at a primary school in Opuwo, in a class of 20 children with learning disabilities. This is the only inclusive school with a special class in Opuwo. These learners were not formally assessed for or diagnosed with a LD by a professional; they were placed in this class because of their poor academic performance. By means of convenience and availability sampling, only eight caregivers volunteered their participation in the study. Participants were purposively selected seeing that they had to be a parent of a child in the class for children with learning disabilities. The sample comprised parents who were willing to participate and who were available to take part in an interview (Kumar 2011). Participant characteristics are displayed in Table 1.

**TABLE 1:** Biographical profile of participants.

Participant	Age	Gender	Marital status	Occupation	Relationship with child	Age of child	Gender of child
1	46 years	Female	Married	Teacher	Foster parent	9 years	Male
2	34 years	Female	Single	Unemployed	Biological parent	10 years	Male
3	37 years	Female	Single	Unemployed	Foster parent	8 years	Male
4	41 years	Female	Divorced	Cleaner	Biological parent	10 years	Male
5	35 years	Female	Single	General worker	Biological parent	10 years	Male
6	44 years	Female	Single	Unemployed	Biological parent	12 years	Male
7	42 years	Female	Married	Teacher	Biological parent	9 years	Female
8	34 years	Female	Single	Unemployed	Biological parent	9 years	Male

*Source*: Authors’ own work

As indicated in Table 1, the mean age of children reported by the caregivers was 9.63 years ($\bar{x}$ = 9.63; SD = 1.19), while the mean age of the participants was 39.13 years ($\bar{x}$ = 39.13; SD = 4.73). The ages of the children ranged from 7 to 12 years. Seven out of eight of the represented children were boys. This was an outcome of convenience and availability sampling, even though both boys and girls were enrolled in the specific class for children with LDs. The table also indicates that six out of eight participants were biological parents, and two participants were foster parents. Of these eight participants, only two were married, one was divorced while the rest were never married. Four out of eight participants were unemployed. All participants were women and were Otjiherero speaking.

Given that the researchers sought to obtain sensitive information regarding challenges faced by parents in parenting children with LDs, unstructured one-to-one interviews were utilised. Greeff (2011) points out that at the root of unstructured interviewing is an interest in understanding the experiences of people and the meaning they ascribe to that experience. To develop insight regarding the main issues that affect parents in parenting children with LDs, the interviews were guided by the following research question: ‘What are the challenges faced by parents in parenting children with learning disabilities?’ The two sub-questions, relating to challenges, were ‘What services and programmes are accessible regarding the parenting of children with learning disabilities?’ as well as ‘How do social workers provide assistance in addressing the challenges faced in parenting children with learning disabilities?’

The researchers utilised the services of an interpreter during four of the interviews where participants could not express themselves adequately in English. Where the participant was not fluent in English, the researcher asked for permission to involve an interpreter. These interviews were then conducted in Otjiherero. The interpreter signed a confidentiality agreement as an assurance of abiding by the principle of confidentiality. All interviews were audio recorded to facilitate transcribing and translation. This aspect was included in the informed consent letters signed by participants.

After transcribing and translating the interviews, the researchers identified, analysed and reported on patterns and themes within the data by means of thematic analysis (Braun & Clarke 2006). Thematic analysis allowed the researchers to describe the data set in rich detail, seeing that this method of analysis involves searching across the data set to find repeated patterns of meaning (Braun & Clarke 2006). To ensure trustworthiness of qualitative research, peer debriefing was conducted with social workers outside the research project who work closely with children with disabilities in the Opuwo district and who have experience of the research population (Lietz, Langer & Furman 2006). Their knowledge of the target population and subsequent input increased the credibility and authenticity of the research. The researchers also kept an audit trail of all the steps, actions and decisions made throughout the research to ensure that the research was logical and well documented (Schurink, Fouché & De Vos 2011).

## Discussion of findings

- Challenges experienced by caregivers based on knowledge and perceptions regarding learning disabilities.
- Challenges faced by participants regarding accessibility of specialised programmes and services for the child with a LD.
- Support received by participants from family and external sources.

### Challenges experienced by caregivers based on knowledge and perceptions regarding learning disabilities

Caregivers demonstrated some challenges in comprehending LDs. Although their children were in the class for learners with learning disabilities, no formal diagnosis regarding the disability was conveyed to any of the participants. This lack of information was confirmed by specifically asking participants to provide information on this aspect. They expressed themselves in the following manner: ‘I think it is when a child cannot cope and his mind is behind that of other learners’ (Participant 1, 46-year-old married foster parent, employed as a teacher). ‘Umm … I think it is when a child has got a problem in thinking and cannot concentrate or listen to what he is told … I don’t know’ (Participant 8, 34-year-old single mother, unemployed).

Participants seemed to rely on casual information from friends and teachers who do not have specific knowledge in the domain of LDs. The following illustrate how some participants were informed about LDs and the services provided:

‘You know that when he was born the pregnancy was only six months and was put in the incubator for three months. So I thought maybe that’s why the mind is not progressing. I said to myself let me take him to the teacher and be admitted in the special class at the junior school. … After three months she told me ooh! The child’s mind is behind the age.’ (Participant 1, 46-year-old married foster parent, employed as a teacher)

‘The teacher then called me and said this child is a problem … we tried and tried but the child is not improving. Why not take this child to special class at the junior school? It’s not like the child is mentally sick he is just fine, he is just ok. We just don’t know why the child goes slowly. I then took him to the junior school.’ (Participant 6, 44-year-old single mother, unemployed)

The above indicate the haphazardness of information regarding LDs. No participant received formal information regarding LDs and the services available. Instead, these caregivers relied on informal sources of information. Even though vague, information from these sources resulted in parents’ growing awareness of their child’s difficulties. Freedman and Boyer (2000) state that the initial attempt to obtain information regarding a child’s disability is a time-consuming and difficult process as caregivers are bound to meet obstacles. These authors conducted a study with 40 parents of children with disabilities in the USA. The parents revealed that access to information is a difficult process as caregivers have to hunt for relevant services and information. Parents of children with LDs in Namibia are no exception to this finding, seeing that Namibia is a developing country with fewer resources and services available than in the USA.

The lack of understanding of LD as a phenomenon is emphasised by Abosi (2007), who argues that in Africa most governments have not considered it worthwhile to invest in the research of LDs to determine their causes and prevalence. Abosi (2007) further mentions that because of lack of a unified assessment process regarding LDs in Africa, there is a vague understanding of the term among the general public. However, participants in this study managed to mention some of the components of LDs such as being unable to cope in class, lacking concentration when listening and being unable to do well in school. These components imply the impairment of cognitive functioning as described by the Department of Health in England, cited by Gobrial (2009).

From a social model of disability perspective, lack of formal communication regarding the disabilities and impairments of children inhibits self-determination and social justice for children with LDs and their families. Proponents of the social model of disability, such as Harries and Enfield (2003), mention that persons with disabilities and where appropriate their families and advocates should have access to full information on diagnosis, rights and available services and programmes. Participants did not perceive LDs as a form of disability but as general poor performance at school caused by certain circumstances. Some participants expressed their views about these circumstances in the following manner:

‘In my understanding my separation with my husband is the contributing factor to his poor intellectual performance because he used to love his father so much and now that love is no more.’ (Participant 4, 41-year-old divorced mother, employed as a cleaner)

‘My child grew up with my mother and I think the way she was brought up is not good because she used to do everything for her, that’s why she cannot perform at school.’ (Participant 6, 44-year-old single mother, unemployed)

These statements indicate that participants realise that their children are not performing adequately on an academic level. However, they regard separation from loved ones and a pampered and spoilt way of upbringing by caregivers as the cause of their child’s poor performance in school. Knowledge and perceptions regarding learning disabilities can be regarded as a challenge, as indicated above. Theme 2 focuses on accessibility of specialised programmes and services for the child with a LD.

### Challenges faced by participants regarding accessibility of specialised programmes and services for the child with a learning disability

Regarding awareness of programmes and services, all participants specified that they were not aware of specific programmes and services for LDs. Usually, programmes for children with learning disabilities that parents need to be aware of relate to medical care, educational support and daily living support. In Opuwo, such parents may approach the Ministry of Gender Equality and Child Welfare for financial assistance if need be; they may engage in CBR initiatives or seek informative counselling from social workers in various government ministries. Freedman and Boyer (2000) state that obtaining accurate and useful information about these services and programmes is a major problem encountered by parents of children with disabilities. The participants expressed the following views: ‘We are struggling you see. I want our government to really assist us in this’ (Participant 7, 42-year-old married mother, employed as a teacher). ‘There are no services or programmes I am currently benefiting from’ (Participant 6, 44-year-old single mother, unemployed). As participants indicated that they were unaware of programmes and services, they also did not have knowledge about relevant policies. Rainey et al. (2003) postulate that addressing socio-economic problems sustainably encompasses a set of policies and activities that work together to create economic vitality and social equity. It seems as if the participants’ lack of knowledge about LDs as a phenomenon results in a lack of knowledge pertaining to the relevant services, programmes and policies. Therefore, based on a lack of information on LDs, almost all participants had limited access to services pertaining to LDs, namely social grants, government educational support and specialists in LDs. Important services and programmes available for children with disabilities, such as family visitors’ programmes, community-focused programmes, mentoring programmes and orphan care programmes that focus on empowering children with disabilities, including those with LDs and their families, were not accessed by the participants seeing that they were unaware of these programmes. Ideally, community-based welfare structures such as CBRC and village health workers assist in initially identifying children with disabilities. The participants were however not familiar with such services. Lack of access to information as well as possibly other access barriers inhibit children with LDs to access essential services such as those mentioned above (Campbell & Oliver 1996; Harries & Enfield 2003). The researchers therefore found that because of specialists not being available and accessible, none of the participants took their children for a formal diagnosis of the LD as within their context they relied on informal information. Although some participants had some idea about specialists, they did not know how to go about consulting such a specialist. These caregivers had the following to say:

‘Last year I wanted to take him to those people who test the mind but I could not get hold of them. I even ask her (*special class teacher*) where I can get those people because I really want to know what is the problem with this kid and until now I did not see them.’ (Participant 1, 46-year-old married foster parent, employed as a teacher)

‘The only challenge I remain with is that I need my child to go through the intelligence tests but I do not know who does those things, that’s my concern.’ (Participant 7, 42-year-old married mother, employed as a teacher)

These statements reveal the struggles and frustrations that participants experience in accessing specialist services. This confirms what Taylor (2005) refers to when mentioning the struggle to access services and that these parents must strive to become problem solvers, companions and disciplinarians in trying to find the most appropriate way of bringing up such a child. Although the Namibian government provides social grants for orphans and vulnerable children and caregivers could have benefited from such services, not all the participants knew about such a government programme (Republic of Namibia 2011). Participants expressed themselves in the following manner: ‘I don’t know of such service, we just struggle with what we have’ (Participant 5, 35-year-old single mother, employed as a general worker). ‘I have tried to get assistance from the government to get money to support this child but nothing came out’ (Participant 8, 34-year-old single mother, unemployed). The social model of disability which is founded on the notion of socio-economic empowerment implies that social and economic structures disable impaired people by excluding them from full participation in mainstream socio-economic activities (Oliver 2004). This is evident among the participants who expressed that they experience financial hardships and are not aware of services and programmes that their children should be benefiting from. The difficulties that caregivers experience in accessing services, which could leave them feeling demoralised, are also confirmed by the findings of Resch et al. (2010:142) in their research in the USA where one of the participants in their study mentioned: ‘We hear so many no, no, no, when we are looking for information; it’s a fight for everything’. With the fragmentation of social services in Namibia, a similar challenge may be experienced by parents of children with LDs (Republic of Namibia 2011).

Access to services regarding LDs was therefore a noticeable challenge, as indicated above. This supports the finding by Booyens et al. (2015) that caregivers in Botswana, Malawi and South Africa experienced difficulties in accessing information and services for children with disabilities. They reported on insufficient and inadequate resources available to children with disabilities and their families, especially those living in rural communities. This challenge is exacerbated by the fragmentation of services as children’s services in Namibia are provided by different ministries (Republic of Namibia 2011), as discussed under the literature review. Saloojee et al. (2007) also referred to fragmented services and parents being ill-informed based on research conducted in Orange Farm in South Africa. Theme 3 focuses on the support received from families and external sources in trying to assist children with LDs, as well as their parents.

### Support received by participants from family and external sources

The children’s caregivers mentioned three levels of support, namely: support from the biological fathers, support from other family members, as well as support from the community. It was however of interest to learn that participants described the support provided by the biological fathers as minimal and support from the community as even less. Some communities in Namibia perceive disabilities as a punishment for what one has done wrong (Chilwalo 2010). This could perhaps explain the minimal support received from the biological fathers of the children with LDs, as well as from the community. Only two participants mentioned the involvement of fathers in raising their children. The other six participants were solely responsible for the well-being of the children. Asked what support they receive from the biological fathers of the children with LD, participants indicated the following:

‘I am not getting support from anyone, the father of the child is not supporting; I am just struggling on my own. I cannot force someone to support the child. I will try to support with the little that I have. Since the birth of this child, the father never supported.’ (Participant 5, 35-year-old single mother, employed as a general worker)

‘I don’t know about the support of the father but I recall one point when he sold a goat and tried to support the child but the money did not reach the child who was staying with the grandmother by that time.’ (Participant 3, 37-year-old single foster parent, unemployed)

‘There is no assistance from the father of the children.’ (Participant 8, 34-year-old single mother, unemployed)

These findings could relate to Chilwalo’s (2010) statement that myths and beliefs about a child with disabilities, such as ‘a curse from God’ and ‘an unfaithful wife’ being the cause of the disabilities, may cause a lack of cooperation from the father of the child and to some extent marriage breakdown. In the context of a patriarchal society like Namibia, where men have decision power, these myths and beliefs result in assigning blame to women (LaFont & Hubbard 2007). Burke (2008) adds that because of the superstitions mentioned above, mothers are in most instances left to bear the brunt of caring for the child with disabilities. Based on their research in Uganda, McNally and Mannan (2013) confirmed this finding and indicated that mothers of children with disabilities were the primary caregivers. In Uganda, rejection by fathers is common. Spousal and immediate family support is therefore absent; the onus of care falls on the mothers (McNally & Mannan 2013). Mudhovozi et al. (2012) also emphasise that in Limpopo province, South Africa, the burden of raising a child with disabilities lies with the mother only.

In this study, many participants did not receive much support from family members for their children with learning disabilities.

‘I don’t have any support from anyone. The only time I used to get support was when my mother was still alive. She could give me some food and money but now, nothing. My uncle just helped me once last week when he saw that the children were hungry. He bought a bag of maize.’ (Participant 8, 34-year-old single mother, unemployed)

‘We are not staying together with family so there is no support from the family’ (Participant 2, 34-year-old single mother, unemployed). ‘Neither the family nor the community assists me in supporting my children’ (Participant 4, 41-year-old divorced mother, employed as a cleaner). These sentiments indicate the challenges that participants experience as the sole caregivers of their children with disabilities. An interesting observation in this study is that participants who were lone parents, who did not receive support from the biological fathers, also mentioned that they were not receiving any support from family members. Family support was mostly rendered to a child of a married couple. This may be influenced by culture, as one participant mentioned that if a ‘Himba’ woman, a culturally rooted ethnic group in the north-west of Namibia, is impregnated by a married man, he will not support the child.

If children from these single parents should have LDs, the possibility that they might suffer a double stigma is increased; hence, little or no visible support is received from family members. The lack of support by family and community members seems to predispose participants to stigma and discrimination (Harries & Enfield 2003; Swain et al. 2004). The third level of support explored during the interviews is community support. The majority of participants did not receive any community support. ‘There is no direct involvement of the extended family and the community because I try to provide what my child needs’ (Participant 7, 42-year-old married mother, employed as a teacher).

‘I do not have any support from anyone or the community. In the community no one helps other people’ (Participant 6, 44-year-old single mother, unemployed). From the findings, it was evident that most participants were not receiving support from the biological father of the child, their family members or the community at large. As no one experienced any form of social work support, most of the participants expressed a need for support from social workers during the interviews.

‘I feel social workers should teach some of us parenting skills because sometimes we spoil them by giving them too much attention. Sometimes I think of doing it the African way, just commanding.’ (Participant 7, 42-year-old married mother, employed as a teacher)

‘The thing is, I am unemployed and I am struggling to support my child. I just wish if the social workers can help me secure a job so that I can fend for her and her siblings.’ (Participant 3, 37-year-old single foster parent, unemployed)

The above statements plainly indicate that these participants rely on their own resources to care for their children with LDs. Jackson and Abosi (2006) mention that the behaviour and practice of almost all people in Africa have been influenced by modernity to some degree but when it comes to an association with children with disabilities, many are highly conservative as they prefer to keep children with disabilities at arm’s length. McNally and Mannan (2013) are in support when they refer to increased isolation experienced by caregivers in Tanzania as supporting children with disabilities is not regarded as the responsibility of the community.

## Conclusions

It became clear from the research that parents of children with LDs face a range of challenges, such as difficulties accessing information, services and programmes. The fragmented nature of these services may add to the challenges experienced in this regard. These findings support those of Abosi (2007), who argues that LDs are often not clearly understood in African contexts (Abosi 2007). Participants were also uninformed regarding essential policies meant to benefit their children with learning disabilities.

It could also be concluded that the participants themselves seem to have stereotypes and prejudices regarding LDs. The majority of fathers, referred to by the caregivers, also experienced prejudices as fathers are thought to be avoiding prejudice by distancing themselves from the children; therefore, they did not take responsibility for the emotional and financial support of these children. Extended family as well as community members also distanced themselves from the caregivers and their children with learning disabilities. Some participants voiced that they wanted to be assisted by social workers in areas of parenting and job opportunities in order to assist their children living with LDs. It is the view of the researchers that social workers may have an opportunity to make a difference in the area of LDs if they empower the caregivers of these children, as well as the children themselves. In the next section, recommendations in terms of improving the situation of parents of children with LDs and their children will be made.

### Recommendations

Social workers as change agents can make a difference in the lives of children with LDs and their families in a number of ways. By using the social model of disability, social workers can address sociopolitical constructs that cause challenges such as the perceptions of and attitudes towards LDs. Seeing that myths and cultural beliefs about learning disabilities exist in the context of Namibia, social workers in particular should, through community awareness, educate communities in terms of LDs, policies regarding LDs as well as special and inclusive education. Social workers may do this by working with community health forums, health extension workers or other established forums. Most importantly, social workers in their role as educators and advocates can help educate communities on the rights of people with disabilities. Areas of focus as stipulated in the Convention on the Rights of Persons with Disabilities are that persons with disabilities deserve to be respected as individuals with inherent dignity; they should not be discriminated against on the basis of their impairments and deserve equality of opportunities (United Nations 2008).

Regarding access to services, practitioners should advocate for the rights of persons with disabilities and their caregivers to access information and other necessary services to meet their needs. Accessibility to information can be enhanced by utilising CBRC as well as the Constituency Child Welfare Forum to disseminate information regarding LDs. This is an appropriate role for social workers, seeing that the core values of the social work profession include service delivery and the enhancement of social justice, dignity and worth of individuals, groups and communities. In Namibia, social workers can embrace their traditional role of assisting beneficiaries (parents of children with LDs and their children) by linking them to resource systems, such as the family visitors’ programme, mentoring programmes, referral to appropriate schools, health services including specialists for LD diagnosis, CBRC as well as other relevant services as per the provision of the IECD. Furthermore, social workers can monitor the delivery of such services through conducting home visits and child welfare forums to ensure that the services are structured, organised and culturally accessible and practised in a way that is in accordance with the human rights convention.

To assist caregivers of children with disabilities who face challenges caused by fragmented services, practitioners can advocate for the unification of these fragmented services and lobby the applicable ministry in Namibia. As participants were not aware of any existing policies pertaining to LDs, it is recommended that social development practitioners embark on wider policy education and mobilisation of grass-roots participation in formulating public policies relating to LDs. Most parents of and children with LDs in this study did not receive support from extended family members and the community. Practitioners can embrace the Ubuntu concept as a framework for mobilising support for children with LDs. Patel (2015) describes Ubuntu as caring for and promoting the human dignity and worth of people. This can be attained by conscientising community structures such as traditional leadership, religious and political leadership on the roles and benefits that families and communities share in terms of collective existence. If a community coexists to support children with disabilities, parents or caregivers could be informed and encouraged to access antenatal services, nutritional services as well as professional services critical to the development of children. This will enable children with learning disabilities to exercise their worth as human beings.

The socio-economic circumstances of most participants posed a challenge in providing for the material welfare of their children with LDs. Therefore, practitioners should empower these caregivers by helping them to access government grants or to establish small enterprises which can generate income that will help them meet their needs seeing that they are often not able to rely on the fathers of their children for emotional and financial assistance. This finding is in support of previous research findings. In order to fill the knowledge gap, further research is required within the Namibian context on the perceptions of community members regarding the phenomenon of LDs. It is also recommended that further research be conducted on a broader target population seeing that this study focused on the Opuwo community only. It will be valuable to explore and describe a cross-section of perceptions regarding learning disabilities within a broader context. Where interpreters are used in future research, the possibility of meaning being distorted by the paraphrasing of words in the process of translation should be addressed in a specific attempt to ensure authenticity. In this research, this could be considered as a possible limitation of the study.

This research confirmed previous research findings regarding stigma related to disability, which results in lack of support by fathers and increased isolation. Parents were not aware of specific programmes and services for vulnerable children, which includes children with LDs. This problem is exacerbated by the fragmentation of services. This highlights the plight of children with disabilities and their parents in impoverished communities such as Opuwo, Namibia. Social workers are positioned to address the needs of these vulnerable service users by means of the strategies discussed.
